# Earth-friendly-assessed silver-nanoparticles spectrophotometric method for rapid and sensitive analysis of Molnupiravir, an FDA-approved candidate for COVID-19: application on pharmaceutical formulation and dissolution test

**DOI:** 10.1186/s13065-023-00933-2

**Published:** 2023-03-10

**Authors:** Ahmed R. Mohamed, Ebrahim Abolmagd, Israa M. Nour, Mohamed Badrawy, Mohamed A. Hasan

**Affiliations:** 1 Analytical Chemistry Department, Faculty of Pharmacy, Egyptian Russian University, Badr City, Cairo, 11829 Egypt; 2 Analytical Chemistry Department, Faculty of Pharmacy, Al-Azhar University, Nasr City, Cairo, 11751 Egypt

**Keywords:** Molnupiravir, COVID-19, Silver-nanoparticles, Dissolution test, Eco-scale, GAPI

## Abstract

Molnupiravir is the first oral direct-acting antiviral prodrug recently approved for the COVID-19 pandemic. Here and for the first time, we present a novel, sensitive, robust, and simple silver-nanoparticles spectrophotometric technique for molnupiravir analysis in its capsules and dissolution media. This spectrophotometric technique involved silver-nanoparticles synthesis through a redox reaction between the reducing agent (molnupiravir) and the oxidizing agent (silver nitrate) in presence of polyvinylpyrrolidone as a stabilizing agent. The produced silver-nanoparticles have an intense surface plasmon resonance peak at 416 nm where the measured absorbance values were utilized for the quantitative analysis of molnupiravir. The produced silver-nanoparticles were recognized by using the transmission electron microscope. Under optimal conditions, a good linear rapport was accomplished between molnupiravir concentrations and the corresponding absorbance values in a range of (100–2000) ng/mL with a detection limit of 30 ng/mL. Greenness assessment was implemented using eco-scale scoring and GAPI disclosing the excellent greenness of the suggested technique. The suggested silver-nanoparticles technique was authenticated according to recommendations of the ICH and statistically assessed with the reported liquid chromatographic method without significant differences regarding accuracy or precision. Accordingly, the suggested technique is deemed a green and cheap alternative for assaying molnupiravir due to its reliance primarily on water. Furthermore, the suggested technique’s high sensitivity can be employed for investigating molnupiravir bioequivalence in future studies.

**Graphical Abstract:**

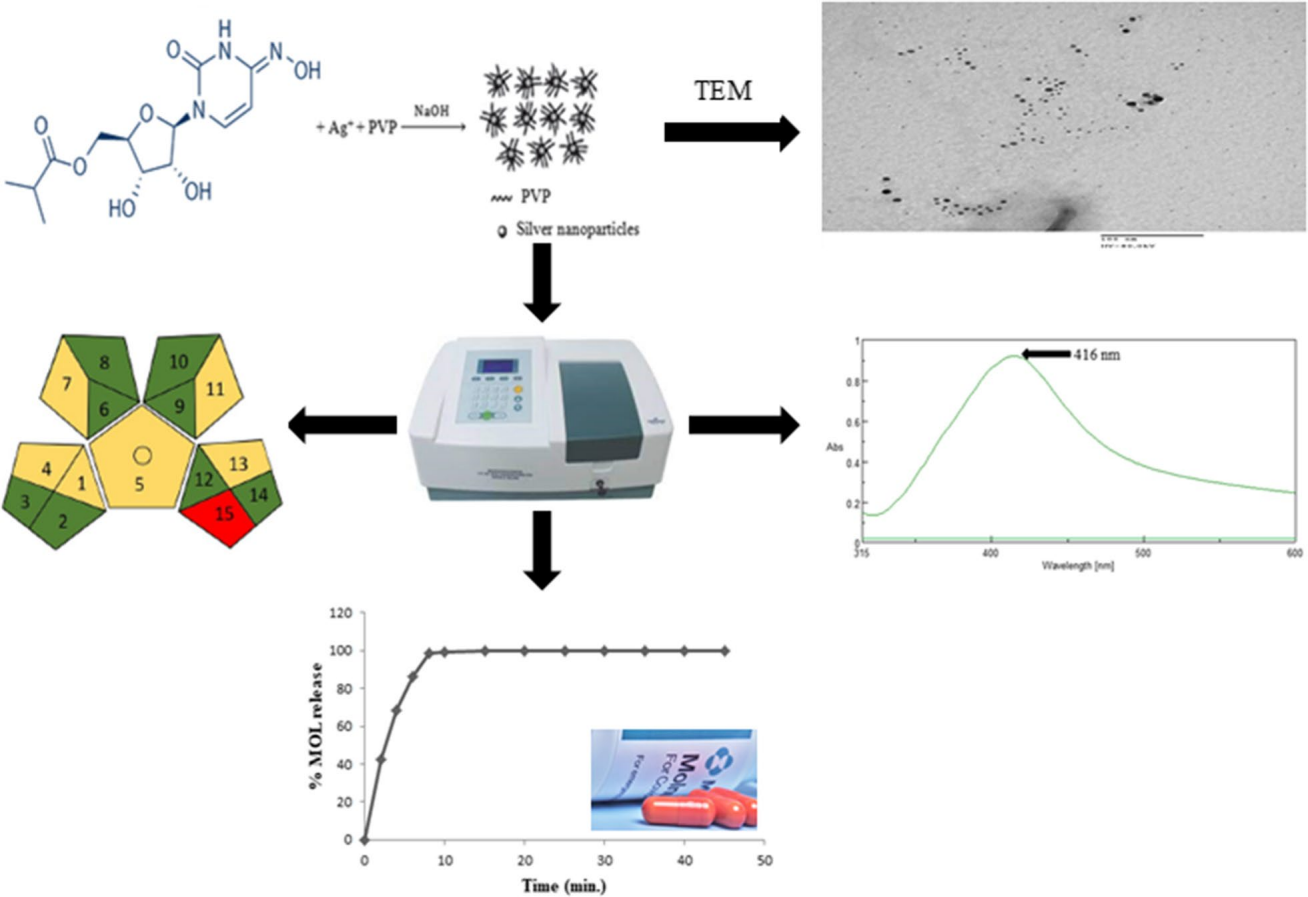

## Introduction

**Fig. 1:**
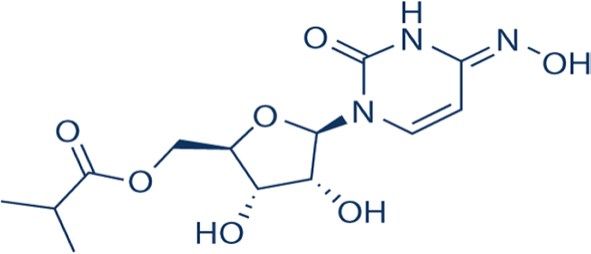
Chemical structure of Molnupiravir

COVID-19 is a pandemic disease that swept the world in 2019 and still infecting millions of people. Many attempts have been made to confront this pandemic, which has led to the death of many people and hindered life in general for others. Coronaviruses including the SARS-CoV-2 virus (the main causative agent of the COVID-19 pandemic) depend on using an RNA-dependent RNA polymerase for their replication and transcription of their RNA genome [1–3]. So, we introduce molnupiravir (MOL) (Fig. 1) as an isopropyl-ester prodrug of the nucleoside analog; β-D-N4-hydroxycytidine (EIDD-1931 or NHC) that targets RNA-dependent RNA polymerase leading to fatal errors in replication of SARS-CoV-2 infectious virus [4–6]. As a recently approved promising medication for COVID-19, MOL is the first oral direct-acting antiviral prodrug shown to be highly efficient at reducing nasopharyngeal SARS-CoV-2 infectious virus as well as viral RNA [6]. MOL has a favorable tolerability and safety profile moreover, it improves the clinical rates of recovery and decreases the length of stay in the hospital [7].

Silver-nanoparticles (Ag–NPs) exhibit extensive antimicrobial activities besides their prodigious uses in numerous fields particularly in the areas of drug delivery and analysis. In the area of drug delivery, Ag–NPs are utilized to guide the drugs to the diseased tissues specifically in the field of chemotherapy, increasing therapeutic effectiveness and reducing potential medication side effects [8]. In the area of drug analysis, Ag–NPs are used to develop numerous sensitive and green approaches at the lowest cost for the determination of drugs due to their major dependence on the water as a cheap and environmentally friendly solvent [9]. Furthermore, Ag–NPs as a promising technique of analysis can determine the analyzed drugs in nano-range and therefore, this advantage was adapted to enhance the proposed spectrophotometric technique’s sensitivity for MOL determination in miscellaneous matrices at ultra-trace concentrations.

According to the literature survey, it was found that only one method was reported for the estimation of MOL [10]. Hence, the purpose of this research is to introduce a novel, simple, cost-effective, and highly sensitive Ag–NPs spectrophotometric technique for MOL rapid analysis in its pharmaceutical formulation and dissolution media without interference by the additives of its formulation yielding satisfactory recovery results relative to those of the reported LC method [10]. Also, this technique is regarded as a green alternative for rapid assay of MOL in its pure form, pharmaceutical formulation, and dissolution media in QC labs laking HPLC due to its dependence primarily on the water as a cheap and eco-friendly solvent relative to the expensive organic solvent utilized in the reported LC method. Furthermore, this technique can be adapted for studying MOL bioequivalence in different biological fluids in future research studies due to the high sensitivity of this proposed technique.

Greenness assessment of the utilized reagents and procedure was performed to evaluate the proposed Ag–NPs technique greenness by using two tools namely; eco-scale scoring and green analytical procedure index disclosing the excellent greenness of the proposed technique.

## Experimental

### Materials and chemicals

All chemicals and reagents used during this experiment were of analytical grade; bi-distilled water was utilized throughout the study.

- The pure standard of MOL was kindly supplied from EIPICO (Sharqia, Egypt) with a purity of 99.89 according to the manufacturer’s purity certificate.
- Methanol, ethanol, acetonitrile, and acetone (Adwic, Egypt).
- Dimethyl sulfoxide (DMSO) and sodium citrate, (Sigma-Aldrich, Egypt).
- Sodium hydroxide, (5 × 10^–3^ M) aqueous solution (Adwic, Egypt).
- Silver nitrate, (2 × 10^–2^ M) aqueous solution (Sigma-Aldrich, Egypt), should be freshly prepared and protected from light during use.
- Polyvinylpyrrolidone (PVP), (0.14%) aqueous solution (Sigma-Aldrich, Egypt).
- Potassium dihydrogen orthophosphate, (5 × 10^–2^ M) aqueous solution (Oxford, India).

Dissolve 20.4 gm of potassium dihydrogen orthophosphate in three liters of bi-distilled water, then pH of the solution was adjusted using sodium hydroxide (to prepare phosphate buffer pH=7.4).

### Pharmaceutical formulation

Molnupiravir^®^ capsules; manufactured by Hetero Corporate, Industrial Estate, India; batch number (HH2112584); labeled to contain 200 mg MOL per capsule.

### Instruments

Jasco model V-630 (Japan) double-beam UV–visible spectrophotometer with two matched 1-cm quartz cells, connected to an ACER compatible PC with spectra manager II software was utilized for measuring the absorbance values over the range (200–800 nm).

A JEOL-1010 transmission electron microscope (Japan) was utilized at 80 kV for transmission electron microscopy (TEM) characterization of Ag–NPs after synthesis et al.-Azhar University.

A USP dissolution type-II (Paddle) apparatus (Model VanKel VK 7000) was utilized for in-vitro dissolution testing.

Sonicator (Model WUC-A06H) and pH meter (Model Jenway 3510) were also utilized.

### Standard solutions

Stock standard solution (100 μg/mL) was made by dissolving 10 mg of pure MOL in 70 mL bi-distilled water into a 100-mL volumetric flask using the sonicator for 5 min. Subsequently, the volume was totaled to the 100-mL mark using the same solvent. Then, a working standard solution (1 μg/mL) was prepared by transferring 1 mL of the stock solution into a 100-mL volumetric flask and then completed to the 100-mL mark with bi-distilled water.

The standard solutions were estimated to be stable for up to 7 days when preserved in the refrigerator as they exhibited no chromatographic or absorbance changes.

## General procedures

### Preparation of Ag–NPs and construction of calibration curve

By using a micropipette, aliquots of MOL were accurately transferred from its working standard solution (1 μg/mL) and followed by the addition of 0.5 mL of AgNO_3_ (2 × 10^–2^ M), 0.7 mL of PVP (0.14%), and 0.5 mL of NaOH (5 × 10^–3^ M) solutions into a series of 10-mL volumetric flasks. Then, the volumes were totaled using bi-distilled water to the 10-mL mark to prepare final concentrated solutions in the range of (100–2000) ng/mL. The prepared solutions were heated for 15 min in a water bath that was thermostatically controlled at 90 °C. After cooling the solutions to room temperature, the absorbance values were recorded at 416 nm versus reagent blank handled similarly and concurrently without MOL (Table 1). Each prepared solution was measured three times. The calibration plot was constructed by relating the absorbance values to the corresponding MOL concentrations in ng/mL followed by computing the regression equation.

**Table 1:** The optimized analytical parameters required for the determination of MOL by the proposed Ag–NPs method

Parameters	Optimized values
λ _max_ (nm)	416
AgNO_3_ (2 × 10^–2^ M) volume (mL)	0.50
PVP (0.14%) volume (mL)	0.70
NaOH (5 × 10^–3^ M) volume (mL)	0.50
Heating time (min)	15
Temperature (°C)	90

Before this procedure, several trials were done on different concentrations of AgNO_3_ (2 × 10^–4^ M–2 × 10^–1^ M), PVP (0.12%–0.15%), and NaOH (5 × 10^–4^ M–5 × 10^–1^ M) using the same concentration of MOL. It was found that the best-concentrated solutions of AgNO_3_, PVP, and NaOH were 2 × 10^–2^ M, 0.14%, and 5 × 10^–3^ M, respectively. Likewise, different volumes were tried for each reagent. It was found that the best volume for AgNO_3_ and NaOH was 0.5 mL while 0.7 mL was the best volume for PVP. Also, the reaction temperature was optimized after several trials at 90 °C for 15 min.

### Application to the pharmaceutical formulation

For dosage form manipulation, the contents of three capsules of Molnupiravir^®^ were weighed, finely pulverized, and homogeneously mixed. An accurately weighed amount of the fine powder equivalent to 10 mg was transferred into a 100-mL volumetric flask and subsequently, the active constituent was extracted with 10 mL bi-distilled water three times using the sonicator for five minutes each time. The solution developed from the extraction process was filtrated into another 100-mL volumetric flask then, the residue was washed several times with 2 mL bi-distilled water. The solution volume was totaled to the 100-mL mark using the same solvent. Afterward, the prepared solution was diluted into a 10-mL volumetric flask using bi-distilled to obtain (1 μg/mL) as a working solution. Finally, the assay was performed as presented before under the general procedure of analysis to compute the nominal content of MOL in its commercial capsules and to apply the technique of standard addition.

### In-vitro dissolution test

The dissolution test was processed on Molnupiravir^®^ (200 mg) capsules using a USP dissolution type-II (Paddle) apparatus that was operated at a rotation speed of 50 rpm for 45 min according to FDA recommendations for dissolution of hard gelatin capsules [11]. The volume of dissolution media required for test performance was one liter of (5 × 10^–2^ M) phosphate buffer (pH = 7.4) that was thermostatically controlled at 37 ± 0.5 ºC. 10 mL aliquots were withdrawn at different time intervals (2, 4, 6, 8, 10, 15, 20, 25, 30, 35, and 45 min), filtered into a series of 25-mL volumetric flasks by using syringe filter (0.45-μm), and subsequently handled as declared under the general procedure of analysis after appropriate dilutions by using phosphate buffer. The withdrawn aliquots were replaced at each time interval with the same volumes of freshly prepared dissolution media. The absorbance values of samples were measured and consequently, the drug release percentage was computed.

### The reported method

LC–MS/MS method [10] was reported for simultaneous analysis of MOL and its metabolite (NHC) in different biological matrices using Atlantis C_18_ column with a gradient elution system of 1 mM ammonium acetate (Amm.Ac) in water (pH = 4.3) (mobile phase A) and 1 mM Amm.Ac in acetonitrile (mobile phase B). The results of the proposed Ag–NPs technique and reported LC method were statistically compared for evaluating the efficiency of the proposed technique.

### Evaluation of method greenness

Two novel approaches were presented to assess the greenness of the proposed Ag–NPs technique with the reported LC method called analytical eco-scale [12, 13] and green analytical procedure index [14].

Analytical eco-scale is a useful semi-quantitative tool employed to evaluate any analytical methodology's greenness. It relies on calculating the penalty points of two main parameters of the analytical procedure. The first parameter is recognized as the reagent parameter which can be estimated by concerning amounts, physical, environmental, and health hazards of the consumed reagents. The second parameter is linked to the instrumentation involving occupational hazards, the instrument's energy consumption, and the amount of waste engendered by the device. After computing the penalty points of the aforesaid parameters, the results are subtracted from 100 to get the total score necessitated for the greenness assessment. The perfect analytical method is granted 100 on the eco-scale score. According to the total score value, the method is viewed as an excellent or acceptable, or inadequate green method.

In Table 2, the calculated penalty points for the proposed Ag–NPs technique were 5 points while were 28 points for the reported LC method revealing the excellent greenness of the proposed technique. Also, these results confirm the preeminence of the proposed technique procedure over the reported LC method due to lower consumption of chemicals and energy as well as lower waste generation.

**Table 2:** Results of eco-scale analysis for the determination of MOL employing the proposed Ag–NPs method and the reported LC method

Methods	Proposed Ag–NPs method	Reported LC method [10]
Parameters		
Reagents		
Methanol	–	12
Acetonitrile	–	4
Ammonium acetate	–	4
AgNO_3_	0	–
PVP	0	–
NaOH	2	–
Phosphate buffer	0	–
Instruments Spectrophotometer/LC–MS/MS		
Energy	0 [≤ 0.1 kWh/sample]	2 [> 0.1 kWh/sample]
Occupational hazard	0	0
Waste	3	6
Total penalty points	Σ 5	Σ 28
Analytical eco-scale total score^a,b^	95	72
	Excellent green analysis	Acceptable green analysis

If the score is > 50, it indicates acceptable green analysis

If the score is < 50, it indicates inadequate green analysis

^a^Analytical eco-scale total score = 100–total penalty points

^b^If the score is > 75, it indicates excellent green analysis

**Fig. 2:**
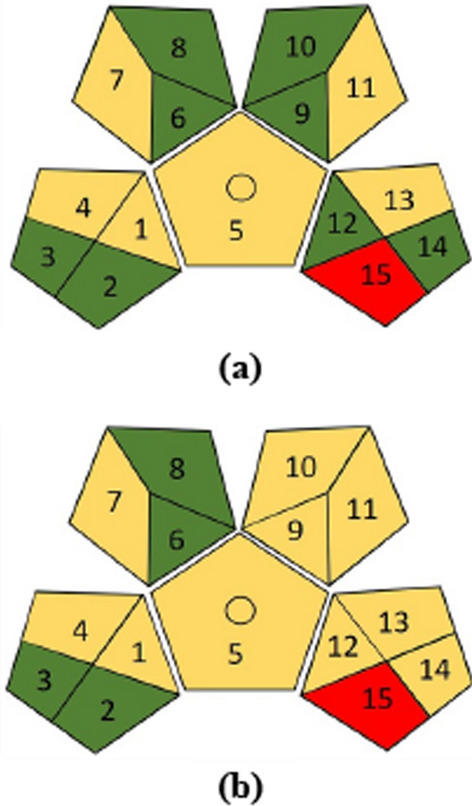
Greenness assessment profiles using the GAPI tool for **a** the proposed Ag–NPs method and **b** the reported LC method during the determination of Molnupiravir

Another tool for greenness assessment of analytical process is green analytical procedure index (GAPI). GAPI is a more advanced tool for greenness assessment. Fifteen-segment pictograms represent different phases of the analytical process from sample preparation to the final detection. Each segment includes three color-specific codes (green, yellow, or red) to indicate the high, medium, or low environmental impact of each step of the analytical methodology. Figure 2a and b showed the greenness assessment profile for the proposed and reported methods’ procedures using the GAPI tool, revealing the preeminence of the proposed technique procedure over the reported method.

Consequently, the proposed Ag–NPs technique excels over the reported LC method as a greener alternative for the quantitative analysis of MOL in its formulation and dissolution media.

## Results and discussion

The COVID-19 pandemic has caused widespread disease and mortality around the world, as well as economic devastation in almost every country. Consequently, MOL has been introduced as the first oral antiviral prodrug that is active against the serious acute respiratory syndrome of SARS-CoV-2. Green chemistry is increasingly being used in laboratories throughout the world for synthesis and analysis to reduce negative environmental effects and increase the health and safety of analysts. As a result, new, environmentally friendly, highly sensitive, and straightforward Ag–NPs technique was introduced for MOL fast analysis in its formulation and dissolution media, producing good recovery results comparable to those of the reported LC method [10].

**Fig. 3:**
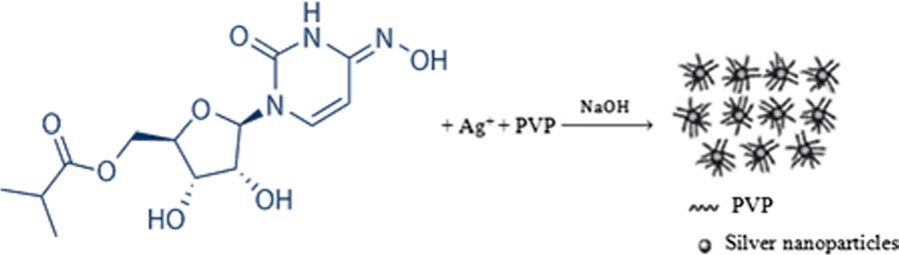
Silver ions reduction by Molnupiravir to stoichiometrically equivalent quantity of Ag–NPs using PVP and NaOH

**Fig. 4:**
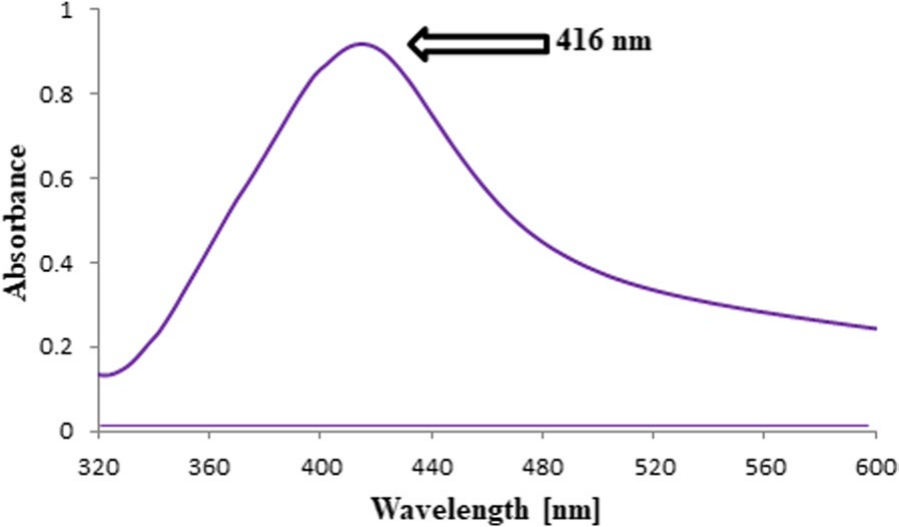
Absorbance spectrum of Ag–NPs developed in presence of Molnupiravir (1400 ng/mL) at 416 nm

**Fig. 5:**
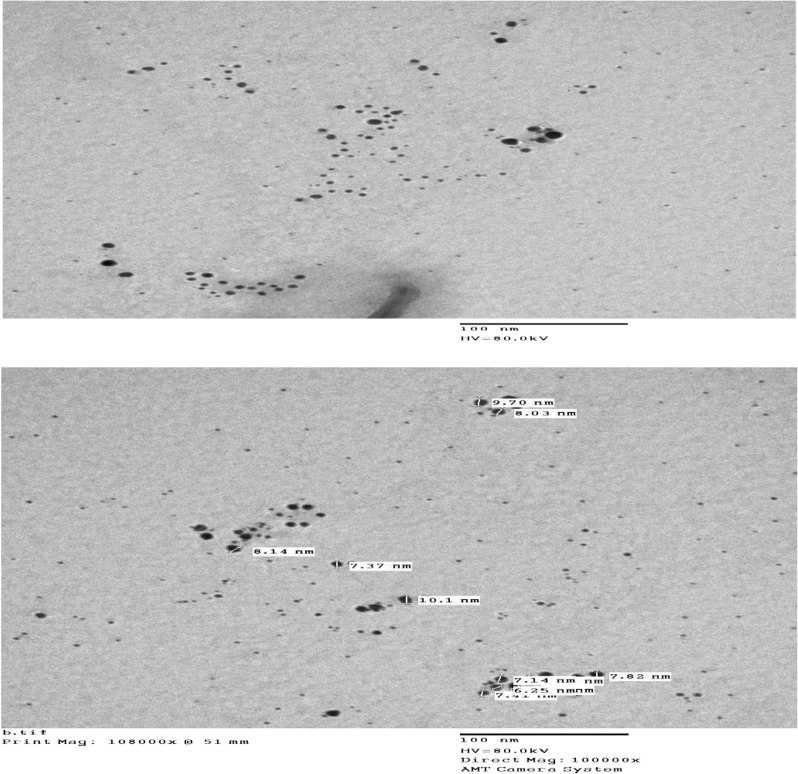
TEM micrograph of Ag–NPs formed in the presence of Molnupiravir

Aqueous AgNO_3_ solution in an alkaline NaOH medium was used in the current reaction system, along with PVP (stabilizer) to prevent Ag–NP agglomeration after their synthesis. Ag^+^ (silver ions) were reduced to a stoichiometric amount of Ag–NPs with fascinating optical characteristics by adding MOL as a reducing agent to the reaction mixture (Fig. 3). The Ag–NPs were distinguished after synthesis by ultraviolet spectrophotometry and TEM. Consequently, the synthesized Ag–NPs exhibited a characteristic absorption peak at 416 nm as the result of the excitation of surface plasmon (Fig. 4). Also, it was noticed that the reaction system failed to produce any observable absorption peaks when MOL was not present in the region (400–700 nm). As presented in Fig. 5, The TEM image confirmed that the formation of Ag–NPs was in presence of MOL and showed that the synthesized Ag–NPs were spherical with smooth surface morphology and size of 8.13 ± 1.67 nm. In contrast to conventional spectrophotometric methods, the proposed Ag–NPs technique was highly sensitive enough to determine very small concentrations of MOL and consequently, can be adapted for the pharmacokinetic study of MOL in biological fluids in future studies.

Conclusively, the proposed method as an eco-friendly technique is appropriate for simple and rapid analysis of MOL in its pharmaceutical formulation at the lowest cost due to its dependence only on the water as a cheap and green solvent.

### Method optimization

To obtain the best possible results of the Ag–NPs approach for the determination of MOL, the following variables were evaluated:

### Influence of concentration and volume of AgNO_3_ solution

**Fig. 6:**
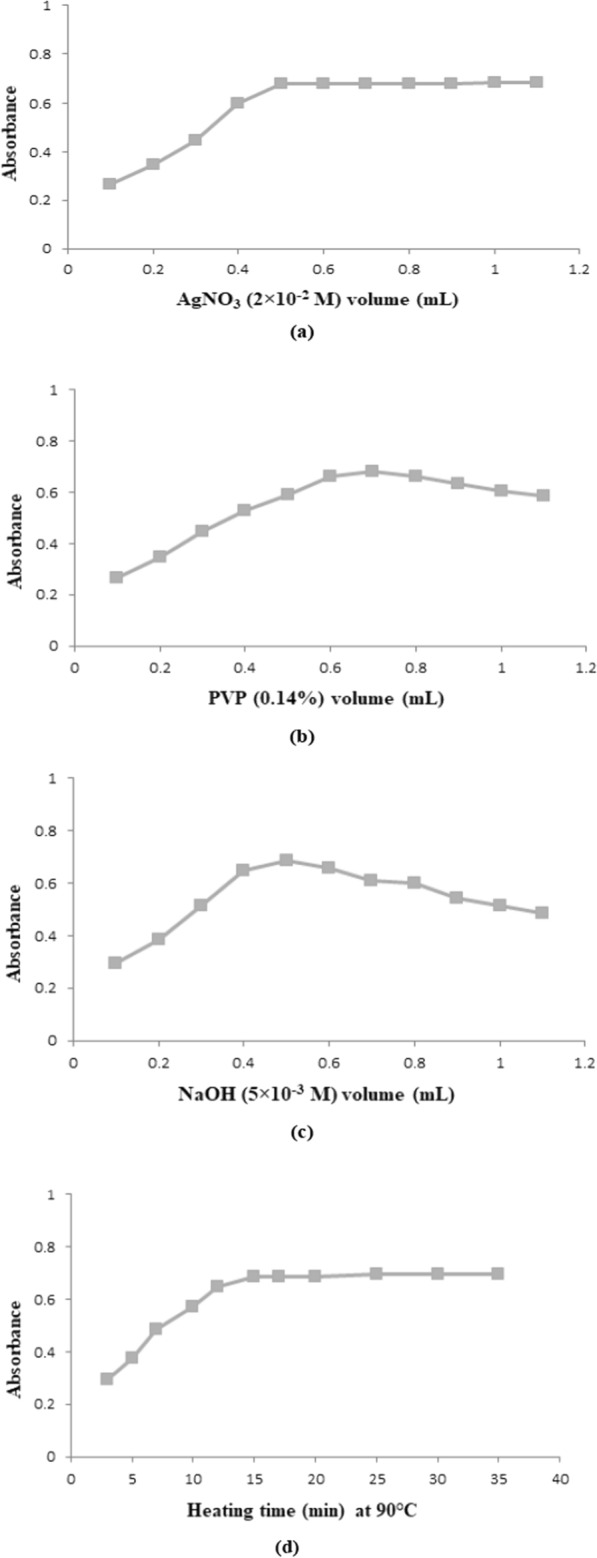
Optimization of Ag–NPs method’s variables using Molnupiravir (1000 ng/mL**)**; including **a** Volume of AgNO_3_ (2 × 10^–2^ M) solution,** b** Volume of PVP (0.14%) solution, **c** Volume of NaOH (5 × 10^–3^ M) solution, and **d** Heating time effect at 90 °C

The procedure was repeated using different AgNO_3_ concentrations with the same concentration of MOL. It was found that the optimum silver nitrate concentration was (2 × 10^–2^ M). Increasing silver nitrate concentration above optimum one caused a significant decrease in the absorbance values of prepared Ag–NPs which affected linearity leading to poor linearity. Then, different volumes of AgNO_3_ solution with concentration (2 × 10^–2^ M) at the other experimental optimum conditions were tried. It was found that 0.5 mL of fixed-concentration silver nitrate (2 × 10^–2^ M) is the optimum volume for the best experimental result. Increasing silver nitrate volume had no effect on the absorbance of Ag–NPs (almost the same absorbance (Fig. 6a).

### Effect of stabilizer type, concentration, and volume

Ag–NPs tend to cluster during their synthesis process. So, stabilizers (electrostatic or steric) must be used during the Ag–NPs synthesis process to prevent Ag–NPs clustering [15]. The mechanism of electrostatic stabilizers (sodium citrate as an example) to prevent nanoparticles’ agglomeration is achieved by causing columbic repulsion between nanoparticles due to the formation of an electrical double layer formed by adsorption of the stabilizers on the nanoparticles’ surfaces. On the other hand, steric stabilizers (PVP as an example) act by forming a protective cap on the surface of nanoparticles preventing their clustering. In this work, PVP was chosen to stabilize Ag–NPs and prevent their clustering due to its higher absorbance values than sodium citrate.

Several experimental trials were done to optimize the concentration of PVP solution. It was found that (0.14%) PVP concentration was the optimum one for the best result. Increasing the concentration of PVP caused a minor decrease in Ag–NPs absorbance and led to poor linearity. Also, different PVP volumes with constant concentration (0.14%) were tested under the same experimental condition. 0.7 mL was found to be the optimum volume which gave the best results. Increasing PVP volume led to a slight decrease in Ag–NPs absorbance (Fig. 6b).

### Effect of concentration and volume of NaOH solution

In process of silver ions reduction to yield Ag–NPs by MOL, a significant amount of H^+^ ions was produced in the medium of the reaction. Buffer solutions failed to keep H^+^ concentration at the level allowed for Ag–NPs formation. So, sodium hydroxide solution was used to solve this problem which provided enough alkalinity to consume the H^+^ ions liberated during the reaction leading to improvement in the reduction process required for Ag–NPs formation. So, different concentrations of sodium hydroxide were tested in a similar way used to test silver nitrate concentration, and found that the best NaOH concentration was (5 × 10^–3^ M). Increasing the concentration of sodium hydroxide caused a significant decrease in Ag–NPs absorbance due to the formation of a black ppt. of Ag_2_O. Also, optimization of sodium hydroxide volume was done using different volumes of NaOH with the same concentration (5 × 10^–3^ M), and found that the optimum volume was 0.5 mL. Increasing NaOH volume led to a gradual small decrease in Ag–NPs absorbance (Fig. 6c).

### Effect of reaction temperature and heating time

It was observed heating is required to complete the process of Ag–NPs synthesis process. The optimum heating temperature was found to be 90 °C in a water bath for a certain time. Rising the reaction temperature above 90 °C resulted in a significant decrease in Ag–NPs absorbance due to the precipitation of silver. Also, different heating times were examined, and found that heating at 90 °C for 15 min was the optimum heating time. Increasing heating time has no effect on Ag–NPs absorbance indicating the end of the Ag–NPs synthesis process (Fig. 6d).

### Effect of diluting solvent

By testing different solvents such as (bi-distilled water, ethanol, acetonitrile, acetone, methanol, and DMSO), it was observed that aqueous media was the best one for optimum absorbance values. Hence, bi-distilled water was the diluting solvent of choice throughout this study. Our technique is therefore more affordable and environmentally friendly than the reported technique.

## Method validation

According to ICH guidelines [16] and the optimized experimental conditions, the proposed green technique was validated yielding satisfactory results.

### Linearity and range

The linearity of the proposed Ag–NPs technique was appraised by analyzing seven concentrations of MOL over the range (100–2000) ng/mL. Also, the calibration graph was constructed by plotting the absorbance values at 416 nm against the corresponding concentrations followed by computing the regression parameters (Table 3). Each prepared concentration of MOL was measured three times.

**Table 3:** Assay parameters for the green analysis of MOL by the proposed Ag–NPs method

Parameters	MOL
Concentration range	100–2000 (ng/mL)
Correlation coefficient	0.9999
Slope	0.0006
Intercept	0.0456
S.D of intercept^a^	0.0056
LOD^b^	30 (ng/mL)
LOQ^b^	90.77 (ng/mL)
Accuracy	
Mean ± SD	100.89 ± 0.60
RSD%	0.60
Er%^c^	0.89
Intra-day precision^d^	
Mean ± SD	101.16 ± 1.12
RSD%	1.11
Er%^c^	1.16
Inter-day precision^e^	
Mean ± SD	100.58 ± 0.95
RSD%	0.94
Er%^c^	0.58

^a^Standard deviation of intercept

^b^LOD = (SD of the response/slope) × 3.3; LOQ = (SD of the response/slope) × 10

^c^Relative error percentage

^d^The intra-day analysis, average of three different concentrations of MOL (500, 1000, and 1500 ng/mL) repeated three times within the day

^e^The inter-day analysis, average of three different concentrations of MOL (500, 1000, and 1500 ng/mL) repeated three times in three consecutive days

### LOD and LOQ

To estimate the proposed Ag–NPs technique sensitivity, LOD and LOQ were computed and listed in Table 3. The exhibited results revealed the high sensitivity of the proposed technique for MOL determination.

### Accuracy and precision

For evaluation accuracy and precision of the proposed method, three different concentrations of MOL were chosen in the linearity range (500, 1000, and 1500 ng/mL) and then determined quantitively in triplicate. Repeatability was performed on the same day while intermediate precision was performed on three consecutive days. The obtained results of accuracy expressed as mean of percentage recoveries showing satisfactory results (Table 3). For repeatability and intermediate precision, values of relative standard deviations (RSD %) were calculated and didn’t exceed 2% revealing the excellent precision of the proposed approach as presented in Table 3.

### Robustness

Robustness of the proposed method was evaluated by changing each parameter of the reaction separately with a small value keeping the other parameters constant. The findings presented in (Table 4) revealed that the proposed technique remained unaffected by the deliberated small changes in the parameters of the reaction indicating the robustness of this method.

**Table 4:** Robustness study of the proposed Ag–NPs method using pure (1000 ng/mL) of MOL

Condition	Results
	Recovery%^a^ ± SD
Optimum condition^b^	99.76 ± 1.12
AgNO_3_ (2 × 10^–2^ M) (0.40 mL)	98.45 ± 0.31
AgNO_3_ (2 × 10^–2^ M) (0.60 mL)	100.61 ± 0.67
PVP (0.14%) (0.60 mL)	99.03 ± 1.08
PVP (0.14%) (0.80 mL)	99.56 ± 0.69
NaOH (5 × 10^–3^ M) (0.40 mL)	98.22 ± 1.30
NaOH (5 × 10^–3^ M) (0.60 mL)	100.34 ± 0.71
Heating time (at 90 °C) (13 min)	98.13 ± 0.44
Heating time (at 90 °C) (17 min)	100.83 ± 0.96

^**a**^Mean of three determinations

^b^Optimum condition as represented in Table 1

## Method application

### Pharmaceutical application

The applied approach was successfully harnessed for quantitative MOL analysis in its commercial Molnupiravir^®^ capsules without pharmaceutical additives interference. The values of percentage recoveries mean and standard deviations presented in Table 5 were agreed to the labeled drug claim. Additionally, the tabulated findings assured that the proposed method was appropriate for the routine analysis of MOL in QC laboratories., especially those lacking HPLC. The proposed technique’s validity was checked by applying the standard addition process yielding good results (Table 5).

**Table 5:** Determination of MOL by the proposed Ag–NPs method in Molnupiravir^®^ capsules and application of standard addition technique

Product	Recovery%^b^ ± RSD	Standard addition			
		Taken (ng/mL)	Added (ng/mL)	Found (ng/mL)	Recovery (%)^c^
Molnupiravir^®^ capsules^a^	100.46 ± 0.76	500	100	99.13	99.13
			300	304.23	101.41
			500	506.95	101.39
			700	698.13	99.73
			900	893.99	99.33
			Mean ± RSD		100.20 ± 1.12

^a^Molnupiravir^®^ capsules, labeled to contain 200 mg of MOL per capsule, batch number (HH2112584)

^b^Mean of five determinations

^c^Mean of three determinations

### In-vitro dissolution test

**Fig. 7:**
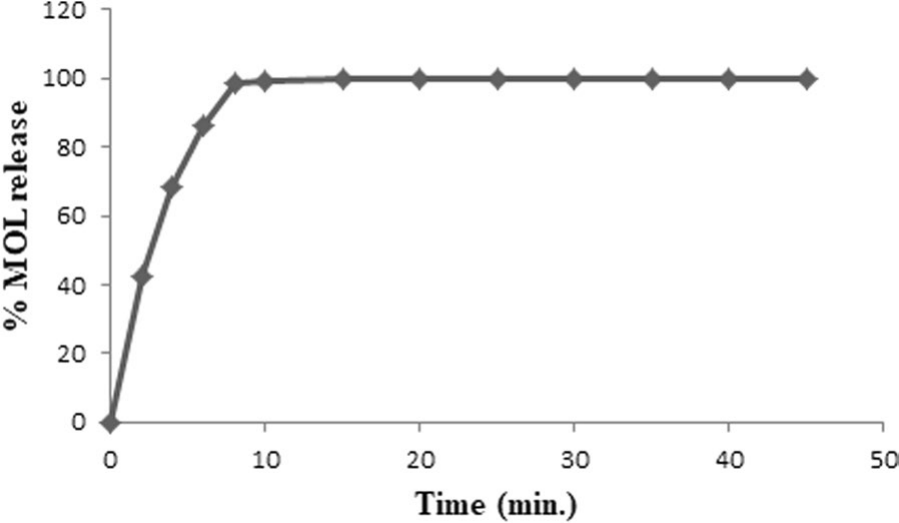
In-vitro dissolution profile of Molnupiravir® capsule using the proposed Ag–NPs method

The dissolution test is crucial for both commercial product quality control and determining how to accurately administer the requisite quantity of the active ingredient (MOL) to patients. The proposed Ag–NPs approach was used to conduct an in-vitro dissolving test on Molnupiravir® capsules to assess MOL release and solubilization from its commercial capsules. Finally, the percentage of MOL release was computed by applying our proposed technique and then plotted versus different time intervals as represented in Fig. 7.

## Statistical analysis

The obtained results (recovery values) by the proposed technique for MOL analysis in its pure form were statistically assessed with those of the reported LC method [10]. Also, *t-* and F- values were calculated (using Microsoft^®^ Excel 2010) and listed in Table 6 where the calculated values didn't outstrip the theoretical ones. According to the statistical comparison in Table 6, no significant differences in results were found between the proposed technique and the reported LC method indicating the high accuracy and precision of the proposed technique (at* p* = *0.05*).

**Table 6:** Statistical comparison between the proposed Ag–NPs and the reported LC [10] methods for MOL analysis in its pure form

Methods	Proposed Ag–NPs method	Reported LC method [10]
Parameters		
Mean	98.89	98.39
SD	0.60	0.26
N	6	6
Variance	0.36	0.07
Student^'^s *t*-test (2.23)^a^	1.87	–
F- value (5.05)^a^	0.19	–

^a^The parentheses contain the corresponding theoretical *t* and F values at (*P* = 0.05)

## Conclusion

It could be deduced from the previous discussion that the proposed Ag–NPs technique is simple, precise, robust, and eco-friendly due to its primary dependence on the water as a green solvent. Because of the attained high selectivity and accuracy by the proposed technique at the lowest cost, this method is regarded to be suitable for routine analysis of MOL not only in QC labs but also for any future research studies with minimum manipulation steps in its pure powder or commercial product. Furthermore, this method is regarded as a cheap and green alternative to the expensive LC methods that utilize hazardous solvents during drug analysis. Also, the high sensitivity attained by the proposed technique can be adapted for studying MOL bioequivalence in different biological fluids in future research studies.

## Data Availability

All data generated or analyzed during this study are included in this published article.

## Abbreviations

MOL: Molnupiravir
EIDD-1931 or NHC: β-D-N4-hydroxycytidine
Ag–NPs: Silver-nanoparticles
DMSO: Dimethyl sulfoxide
NaOH: Sodium hydroxide
AgNO_3_: Silver nitrate
PVP: Polyvinylpyrrolidone
TEM: Transmission electron microscopy
Amm.Ac: Ammonium acetate
GAPI: Green analytical procedure index
Ag^+^: Silver ions
RSD %: Relative standard deviations

## References

[1] Hilgenfeld R, Peiris M (2013). From SARS to MERS: 10 years of research on highly pathogenic human coronaviruses. Antiviral Res.

[2] Dolgin E (2021). The race for antiviral drugs to beat COVID-and the next pandemic. Nature.

[3] Jiang Y, Yin W, Xu HE (2021). RNA-dependent RNA polymerase: Structure, mechanism, and drug discovery for COVID-19. Biochem Biophys Res Commun.

[4] Sheahan TP, Sims AC, Zhou S (2020). An orally bioavailable broad-spectrum antiviral inhibits SARS-CoV-2 in human airway epithelial cell cultures and multiple coronaviruses in mice. Sci Transl Med.

[5] Agostini ML, Pruijssers AJ, Chappell JD (2019). Small-molecule antiviral β-d-N 4-hydroxycytidine inhibits a proofreading-intact coronavirus with a high genetic barrier to resistance. J Virol.

[6] Fischer WA, Eron JJ, Holman W (2022). A phase 2a clinical trial of molnupiravir in patients with COVID-19 shows accelerated SARS-CoV-2 RNA clearance and elimination of infectious virus. Sci Transl Med..

[7] Imran M, Arora MK, Asdaq SMB (2021). Discovery, development, and patent trends on Molnupiravir: a prospective oral treatment for COVID-19. Molecules.

[8] Austin LA, Mackey MA, Dreaden EC (2014). The optical, photothermal, and facile surface chemical properties of gold and silver nanoparticles in biodiagnostics, therapy, and drug delivery. Arch Toxicol.

[9] Tashkhourian J, Hormozi-Nezhad MR, Khodaveisi J (2011). Application of silver nanoparticles and principal component-artificial neural network models for simultaneous determination of levodopa and benserazide hydrochloride by a kinetic spectrophotometric method. Spectrochim Acta Part A Mol Biomol Spectrosc.

[10] Amara A, Penchala SD, Else L (2021). The development and validation of a novel LC-MS/MS method for the simultaneous quantification of Molnupiravir and its metabolite ß-d-N4-hydroxycytidine in human plasma and saliva. J Pharm Biomed Anal.

[11] FDA-recommended dissolution methods; 2023. https://www.accessdata.fda.gov/scripts/cder/dissolution/.

[12] Sayed RA, Mohamed AR, Hassan WS (2022). Earth-friendly-assessed chromatographic platforms for rapid analysis of sulfacetamide sodium and prednisolone acetate in presence of sulfanilamide impurity: application on ophthalmic formulation and aqueous humor. Sustain Chem Pharm.

[13] Gałuszka A, Migaszewski ZM, Konieczka P (2012). Analytical eco-scale for assessing the greenness of analytical procedures. Trends Analyt Chem.

[14] Płotka-Wasylka J (2018). A new tool for the evaluation of the analytical procedure: green analytical procedure index. Talanta.

[15] Sayed RA, Elmasry MS, Hassan WS (2020). The use of surface plasmon resonance band of green silver nanoparticles and conductometry for quantitative determination of minor concentrations of doxycycline hyclate and oxytetracycline HCl in pure and pharmaceutical dosage forms. Int J Nanomanuf.

[16] ICH. Q2A (R1). Validation of analytical procedures: text and methodology. In: International Conference on Harmonization. Geneva: IFPMA; 2005.

